# Superrepellent Porous Polymer Surfaces by Replication from Wrinkled Polydimethylsiloxane/Parylene F

**DOI:** 10.3390/ma15227903

**Published:** 2022-11-09

**Authors:** Fadoua Mayoussi, Ali Usama, Kiana Karimi, Niloofar Nekoonam, Andreas Goralczyk, Pang Zhu, Dorothea Helmer, Bastian E. Rapp

**Affiliations:** 1 Laboratory of Process Technology, NeptunLab, Department of Microsystem Engineering (IMTEK), University of Freiburg, 79110 Freiburg im Breisgau, Germany; 2 Freiburg Materials Research Center (FMF), University of Freiburg, 79104 Freiburg im Breisgau, Germany; 3 Freiburg Center of Interactive Materials and Bioinspired Technologies (FIT), University of Freiburg, 79110 Freiburg im Breisgau, Germany

**Keywords:** surface patterning, polydimethylsiloxane, superhydrophobicity, porous materials

## Abstract

Superrepellent surfaces, such as micro/nanostructured surfaces, are of key importance in both academia and industry for emerging applications in areas such as self-cleaning, drag reduction, and oil repellence. Engineering these surfaces is achieved through the combination of the required surface topography, such as porosity, with low-surface-energy materials. The surface topography is crucial for achieving high liquid repellence and low roll-off angles. In general, the combination of micro- and nanostructures is most promising in achieving high repellence. In this work, we report the enhancement of wetting properties of porous polymers by replication from wrinkled Parylene F (PF)-coated polydimethylsiloxane (PDMS). Fluorinated polymer foam “Fluoropor” serves as the low-surface-energy polymer. The wrinkled molds are achieved via the deposition of a thin PF layer onto the soft PDMS substrates. Through consecutive supercritical drying, superrepellent surfaces with a high surface porosity and a high water contact angle (CA) of >165° are achieved. The replicated surfaces show low roll-off angles (ROA) <10° for water and <21° for ethylene glycol. Moreover, the introduction of the micro-wrinkles to Fluoropor not only enhances its liquid repellence for water and ethylene glycol but also for liquids with low surface tension, such as *n*-hexadecane.

## 1. Introduction

Inspired by the lotus leaves and their natural repellent structures, superhydrophobic surfaces with high water repellence have attracted broad attention from both fundamental research and industrial application perspectives with their applications in various fields, such as self-cleaning [1], drag reduction, anti-icing [2], antifouling [3], anti-corrosion [4,5], and water oil separation [6,7]. Superhydrophobic surfaces are typically identified with a high contact angle (CA) of >150° and a low roll-off angle (ROA) and are achieved by combining the required surface topography (surface roughness with a combination of micro- and nanoscale) with low-surface-energy materials. The repellence properties result from the air pockets trapped in the micro-/nanostructure, which prevents liquids from spreading and reduces the contact between the surface and the liquid. This is known as the Cassie–Baxter state [8,9]. Numerous approaches to fabricate superhydrophobic materials have been reported. These include plasma treatments [10], self-assembly [11], chemical vapor deposition [12], layer-by-layer procedures, sol-gel methods [13,14], and lithography [15]. However, these approaches have many drawbacks as they are typically sophisticated techniques requiring multi-step procedures, featuring long fabrication times and producing fragile hierarchical-surface-roughness structures. Porous materials with bulk porosity were presented to overcome the drawback of fragility. When the micro-/nanostructure is introduced throughout the bulk, the superhydrophobicity is preserved after abrasion, as the material removal exposes a similar surface structure [16,17]. Fluoropor, which was pioneered by our group, is a micro-/nanoporous fluoropolymer with a bulk porosity that makes it insensitive to abrasion [18,19,20,21,22]. The bulk porosity is achieved by using a porogen mixture that contains an emulsifier and a nonsolvent. Fluoropor is a superhydrophobic material with a large contact angle (CA) > 163° for water. Achieving an open, porous, superhydrophobic surface requires supercritical drying of the material, as capillary forces lead to the closing of small nanopores upon drying [23,24,25]. Due to surface tension effects, very small capillary structures such as pores can close up upon drying of the material [23,25]. Therefore, the porosity of the material has to be exposed by opening the surface, which is usually achieved by mechanical force, such as sandpaper treatment [18] or peeling-off using adhesive tape [26]. Recently, supercritical drying has emerged as a valuable technology for retaining the porous structure without the need for mechanical abrasion [27,28,29]. The open porous structure on the surface is crucial for generating the required roughness. On these rough surfaces, liquid droplets will rest on the outermost material points, which retain an air layer between the drop and the substrate, thereby giving rise to the repellent effect. The most stable effect can be obtained by combining micro- and nanostructured substrates, i.e., pores in the corresponding length scales. The influence of the surface roughness on wetting has been thoroughly studied in the past decades [9,26,30,31,32,33]. A concise summary of the physics of wetting was reported in a review of Quéré [34]. Moreover, the introduction of a second roughness hierarchy with features larger than the micro/nanostructure enhances the liquid repellence as it reduces the solid–liquid interface [21,35,36].

Several methods can be used to introduce a structure on a surface; one of the most common strategies is direct replication. Replication relies on the use of templates to create replicates having the desired micro-/nanostructure. In addition to its ease, direct replication reduces the fabrication time, ensures reproducibility, and is also a method widely acceptable in industry as it offers the possibility of mass production. Numerous replicated structures have been reported to fabricate superhydrophobic surfaces. These include pillars [37,38], re-entrant structures [39], and wrinkled structures [40]. Due to its low cost, versatility, and the ability to realize large area patterns, surface wrinkling has shown many advantages in comparison to conventional processes. Wrinkled surfaces are widely used in flexible electronics [41,42] for generating surfaces with special wetting properties and in medicine [43,44,45]. The wrinkling arises when a bilayer system (thin rigid film and elastomeric substrate) is exposed to an extrinsic or intrinsic stress, for example, swelling, thermal expansion, vacuum expansion, or mechanical stretching. Once relieved from the applied stress, surface compression occurs, generating the wrinkled structures [46,47,48]. These vary from simple periodic layers to complex patterned structures depending on many parameters such as the substrate softness, the thickness of the top layer, and the applied stress. Wrinkled surfaces are frequently generated by chemically modifying the surface of a soft material such as PDMS, e.g., by deposition of metals [49], oxidation via exposure to oxygen plasma [50], UV/ozone treatment [51,52], the focused ion beam [53,54], or through vapor deposition of a rigid film (such as hydrocarbon or fluorocarbon) onto a soft substrate [55,56,57]. One of the mostly recent used rigid films are parylene thin films [42,58]. 

Here, we present the fabrication of superhydrophobic surfaces with a stable open porous network by direct replication from wrinkled molds. To prepare the wrinkled molds, PDMS substrates with different softness were prepared and coated with a thin layer of a fluorinated parylene, i.e., PF (37 ± 10 nm), by chemical vapor deposition (CVD). The softness of PDMS substrates was in the range of 7–57 kPa and was easily adjusted by varying the amount of the crosslinking agent. The generated wrinkles showed different wrinkle dimensions/geometries, which resulted from the different PDMS softness. The softer the PDMS was, the longer the wrinkle’s wavelength and the lower the amplitude of the surface topography.

The wrinkled superhydrophobic Fluoropor replicas showed high repellence with a CA of >165° and a roll-off angle (ROA) < 10° for water. When supercritically dried, the porous network at the surfaces was preserved and the surfaces showed a directly exposed micro-/nanostructure (open surface porosity), and no additional surface treatment was required to expose the micro-nanoporous structure of the replicated surfaces. The introduction of surface wrinkles significantly enhanced the liquid repellence of Fluoropor surfaces. 

## 2. Materials and Methods

### 2.1. Materials

(Vinylmethylsiloxane)-dimethylsiloxane copolymer trimethylsiloxane terminated (XG 0677), poly(dimethylsiloxane) vinyldimethylsiloxy terminated (DMS V31), (Methylhydrosiloxane) dimethylsiloxane copolymer trimethylsiloxy terminated (HMS 151), and platinum-divinyltetramethylsiloxane complex in xylene (SIP 6831.1 4) were purchased from abcr (Karlsruhe, Germany). SF00 2k-Silikon (SF) was purchased from SILIKONFABRIK (Bad Schwartau, Germany). Parylene F dimer (PF) was purchased from Fluorochem (Glossop, UK). Ethylene glycol, diiodomethane, *n*-hexadecane, and phenylbis(2,4,6-trimethylbenzoyl) phosphine oxide (PPO) were purchased from Sigma Aldrich (Taufkirchen, Germany). Fluorolink MD700 (MD700) was purchased from Acota (Oswestry, UK). 1*H*,1*H*,2*H*,2*H*-perfluorooctanol (13FOOl) was purchased from BLDpharma (Kaiserslautern, Germany). Cyclohexanol was purchased from Carl Roth (Karlsruhe, Germany). 

### 2.2. Methods

**Scheme 1:**
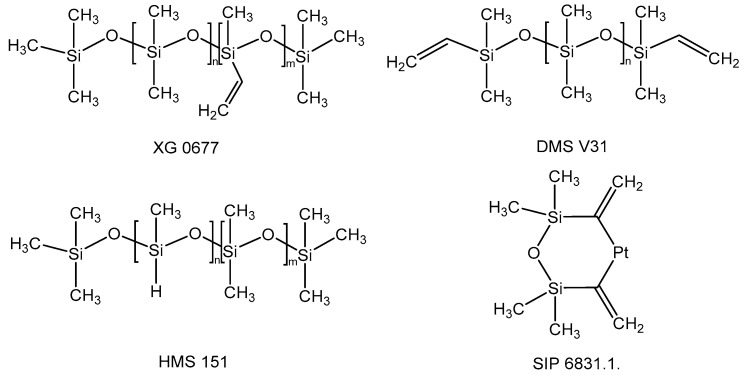
Chemical structure of the chemicals used to prepare Bioclear PDMS.

Preparation of soft PDMS coatings. Bioclear soft substrates were prepared following the procedure reported by Lovchik et al. [59]. The vinylsiloxane prepolymer base (XG 0677, DMS V31) was blended with the catalyst and mixed vigorously. The hydrosiloxane curing agent (HMS 151) was then added to the mixture. All the mixtures contained 5 μL of the catalyst per 5 g of the PDMS (see Table 1). The mixture was then mixed vigorously, and the created air bubbles were degassed under vacuum. The chemical structures of the used prepolymer base, curing agent and catalyst are shown in Scheme 1. Soft PDMS substrates using commercially available two-component PDMS SF was prepared by blending the pre-polymer base and curing agent at ratios of 50:1 by weight (pre-polymer base: curing agent) The mixture was degassed under vacuum. PDMS films with a thickness of 50 µm were prepared via spin-coating onto glass slides. Thicker PDMS films with a thickness of 250 µm were prepared via the casting method.

Softness measurements. The elastic modulus of the PDMS substrates was measured via shear rheology measurements using the HAAKE Modular Advanced Rheometer System of type MARS 2 (Thermo Scientific, Dreieich, Germany). For this purpose, PDMS discs of 2 mm in thickness and 36 mm in diameter were prepared, and the shear elastic modulus (G) was measured to a strain of 0.1% in a frequency range of 0.01 to 10 Hz at 23 °C. 

Chemical Vapor Deposition (CVD) of Parylene. PF thin layers were deposited on the prepared soft PDMS substrates using a parylene deposition System of type PDS 2010 Labcoater (Specialty Coating System (SCS), Woking, UK). The vaporizer was set to a temperature of 150 °C and the pyrolysis oven was set to 650 °C. The deposition of PF on the PDMS substrates was conducted at room temperature and a pressure of 8–15 mbar.

Optical Microscopy: Optical microscopy was performed using a microscope of type VHX 6000 (Keyence Corporation, Japan) with a 20–100 magnification lens. 

White-Light Interferometry (WLI). The thickness of the thin PDMS coatings, surface morphology, and roughness was measured using a White-Light Interferometer (WLI) of type NewView 9000 (Zygo, Middlefield, CT, USA).

Ellipsometry. The thickness of the deposited PF layers was determined using an ellipsometer of type SE 400adv (Sentech Instruments GmbH, Berlin, Germany). For this, PF was deposited on silicon wafers by CVD.

Wetting characterization. CA and ROA measurements were performed using the optical contact angle measurement system OCA 15 (Data Physics, Charlotte, NC, USA). Amounts of 5 μL and 10 μL of liquid droplets were used to measure the CAs and ROA, respectively. For the dewetting behavior characterization, the tilting was set at a speed of 1.24°s^−1^. The average of three measurements was used for both CA and ROA values. 

Preparation of Fluoropor mixture. MD700 (30 wt%) was blended with a porogen mixture consisting of an emulsifier 13FOOl (35 wt%) and a nonsolvent cyclohexanol (35 wt%). The monomer–porogen mixture was then blended with 2 wt% of the photoinitiator PPO.

Direct replication. Fluoropor 35-70 replicas (with 70 referring to the porogen ratio in the mixture and 35 referring to the amount of the nonsolvent in the porogen mixture) were fabricated by pipetting the mixture onto the smooth PDMS and PF-PDMS molds. For comparison, a standard surface was prepared via open-air polymerization. The Fluoropor 35-70 mixture was pipetted onto a supporting substrate. The samples were cured for 5 min (λ = 360–400 nm) with the Hellas UV exposure unit (Bungard, Germany). After the polymerization, the replicas were peeled off the master molds and immersed in acetone for 24 h for cleaning.

Supercritical drying. Fluoropor 35-70 replicas and the standard open-air polymerized surface were supercritically dried to avoid the collapse of the nanoporous structures. The samples were immersed in acetone for 24 h and then transferred to the chamber of the supercritical drying system (Leica EM CPD300). Acetone was replaced by liquid CO_2_ by repeatedly flushing with liquid CO_2_ and releasing the acetone. Afterward, the chamber temperature and pressure were increased to 35 °C and 72 bar, respectively, to maintain the CO_2_ in supercritical condition. Finally, the chamber pressure was dropped gradually to atmospheric pressure to release the CO_2_. 

Scanning electron microscopy (SEM). Surfaces to be analyzed were first sputtered with a 10–20 nm gold layer under an argon atmosphere and then visualized by SEM of type Tescan Amber X (Tescan, Dortmund, Germany). The surface porous area was processed by image analysis using Image-J. The SEM images were loaded into Image-J, followed by adjusting the threshold until the voids/pores were completely outlined. The binary image was then analyzed using the function “analyze particles” and the porosity percentage was calculated (See Figure A3). 

## 3. Results and Discussion

### 3.1. Soft PDMS Coatings

Soft PDMS coatings were prepared using self-mixed Bioclear [59] and commercially available two-component PDMS (SF). The softness of all PDMS coatings was determined by shear rheology measurements. Bioclear was prepared in two different softnesses by varying the amount of crosslinking agent. 

Bioclear B substrates were successfully prepared following Lovchick et al. by blending the low-vinyl-content (XG0677) and the vinyl-terminated dimethylsiloxane (DMS V31) with the low-functionality hydrosiloxane component (HMS151) (see Table 1). The resulting substrate showed a Young’s modulus of 56 ± 6 kPa. Bioclear C was prepared using only XG0677 and HMS151, which resulted in a reduced Young’s modulus of 14 ± 1 kPa. SF (50:1) showed the lowest modulus (7 ± 0.2 kPa), which was achieved by varying the pre-polymer-to-crosslinker ratio.

### 3.2. Properties of Parylene-F-Coated Wrinkled PDMS Thin Layers

To achieve wrinkled surfaces, PF was deposited onto the PDMS surfaces via CVD. The deposited layer shows a thickness of 37 ± 10 nm as determined by ellipsometry. Standard PDMS surfaces exhibit a smooth flat surface, while the substrates coated with PF (PF-PDMS) show a wrinkled surface. The formed wrinkles on the surfaces are a primary result from the difference between the elastic modulus of PDMS and PF. This isotropic wrinkling type often occurs in thermal processes [46]. During the deposition process, the decrease in the vacuum pressure leads to the substrates expansion and the PF thin layer is then deposited onto the expanded substrate. Once the process is finished and the deposition chamber is vented, the substrates contract and generate a large stress at the interface, which creates the surface wrinkles. The compression seemingly points orthogonal onto the substrate edges, leading to this random morphology of the wrinkles with no preferred direction. 

**Figure 1:**
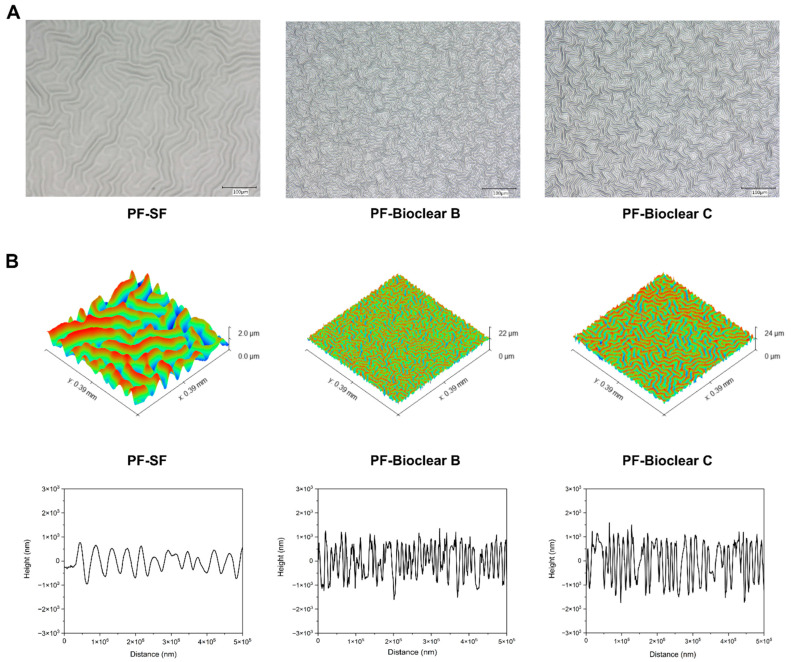
Visualization of the wrinkles on 50 µm PDMS layers. (**A**) Optical microscopy images of the generated wrinkles on SF, Bioclear B, and Bioclear C. (**B**) WLI image of the generated wrinkles and their corresponding profile. SF shows wider yet smaller wrinkles in comparison to Bioclear, which shows thinner and elongated wrinkles.

**Figure A1:**
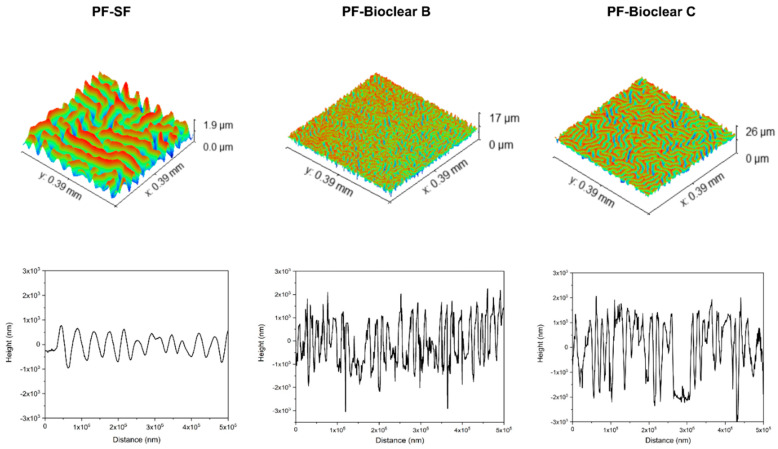
WLI image of the generated wrinkles and their corresponding profile on the 250 µm PF-PDMS films.

The generated wrinkles differ in terms of their periodicity and amplitude. PF-SF shows wrinkles with a longer period and lower amplitude, whereas both Bioclear types show a shorter wrinkle’s period and higher wrinkle amplitude. A decrease in the material’s softness results in larger lateral wrinkle dimensions and a decrease in wrinkle depth (see Figure 1). To quantify the formation of wrinkles, R_q_ values were determined. It was observed that harder PDMS types show an increase in the surface roughness as expected from the images in Figure 1 (see Table 2). It is well-known that material moduli are dependent on material thickness [60,61]. Therefore, the influence on the PDMS thickness on the wrinkle formation was tested with layers of two different thicknesses of 50 µm and 250 µm. WLI images of the generated wrinkles on 250 µm PDMS layers are shown in Figure A1. As can be observed from Table 2, the R_q_ of very soft SF was not influenced by the layer thickness, whereas the harder Bioclear PDMS types showed a decrease in R_q_ values when the thickness was increased from 50 to 250 µm This indicates a change in wrinkle structure for the Bioclear PDMS types upon a change in thickness. 

### 3.3. Superrepellent Wrinkled Porous Polymers

**Figure A2:**
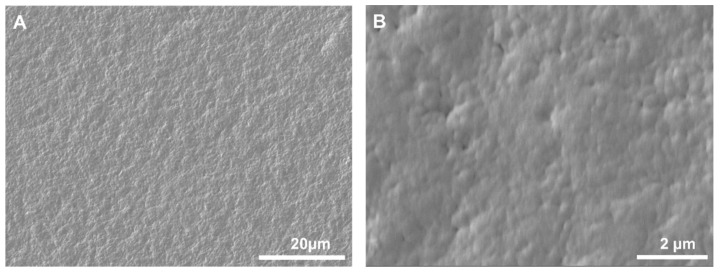
SEM image of an air-dried Fluoropor 35-70 surface. When dried in air, the surface porous network collapses due to capillary forces, which results in a nonporous/closed surface. Scale bar: (**A**) 20 µm, (**B**) 2 µm.

The preparation of repellent surfaces was achieved based on a modified preparation of previously reported Fluoropor foams [18,19,20,21,22]. Fluoropor is based on fluorinated methacrylates mixed with porogens. During the polymerization, Fluoropor forms a highly crosslinked polymer network with a micro-/nanostructure, which is achieved due to a phase-separation of the mix during the polymerization process. The micro-/nanostructure is distributed throughout the bulk. For the purpose of polymer replication, a novel Fluoropor mixture was formulated, which we term Fluoropor 35-70. In this formulation, the porogen amount was enhanced compared to previously reported materials to ensure the achievement of an open porous surface. Fluoropor foams have to be dried after polymerization to remove the solvents. When dried in air, the pores at the surface collapse due to surface tension effects, resulting in a nonporous surface (see Figure A2). To prevent this, supercritical drying was used. The drying process took about 3 h.

**Figure 2:**
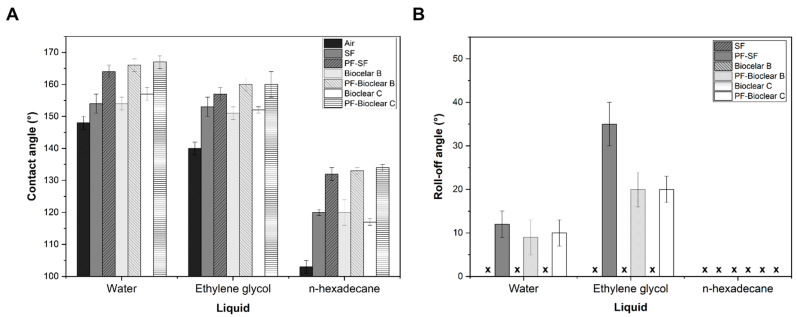
Wetting and dewetting properties of the Fluoropor 35-70 surfaces prepared by open-air polymerization (Air), replicated from smooth PDMS surfaces (SF, and Bioclear B and C), and replicated from wrinkled PF-PDMS surfaces (PF-SF, PF- Bioclear B, and PF-Bioclear C). (**A**) Contact angle values of water, ethylene glycol, and n-hexadecane on the open-air-polymerized Fluoropor 35-70 surfaces (standard), replicas from the smooth PDMS surfaces (SF, Bioclear B, and Bioclear C), and replicas from the wrinkled PF-PDMS surfaces (PF-SF, and PF-Bioclear B and C). The introduction of the wrinkles onto the Fluoropor surface resulted in an increase in the CA of all liquids. (**B**) Roll-off angle values of water, ethylene glycol, and n-hexadecane on the Fluoropor 35-70 surfaces replicated from smooth PDMS and PF-PDMS. X refers to pinned drops with no roll-off angles. The values shown are mean values of 3 measurements and the standard deviation.

Prior to the replication process, the surface free energy of the fabricated PF-PDMS molds was calculated (see Table A1). It is well known that a similarity in the surface free energy of the micro-mold and the replicated material can lead to filling the small features of the mold. However, the fabricated PF-PDMS have low-aspect-ratio micro-wrinkles; thus, no complication in the replication is noticed. To test the effect of the wrinkle structure on the wetting properties of the Fluoropor 35-70 surface, the wetting properties of different surfaces were analyzed (see Figure 2) on open-air-polymerized surfaces, surfaces replicated from soft PDMS, and surfaces replicated from wrinkled PF-PDMS structures. The open-air-polymerized surface was prepared by pouring the Fluoropor 35-70 mixture onto a PDMS mold frame. The wetting and dewetting behaviors were characterized using water, ethylene glycol, and *n*-hexadecane for all surfaces prepared. The three liquids were chosen due to the difference in their surface tension. The wetting properties of a porous Fluoropor 35-70 surface generated under air polymerization show CAs of ~148° for water, ~140° for ethylene glycol, and ~103° for *n*-hexadecane. The three liquids pin on the surface and show no ROAs. 

The Fluoropor 35-70 replicated from soft, smooth PDMS surfaces of three different PDMS types (SF, Bioclear B, and Bioclear C) results in CAs of ~157° for water, ~153° for ethylene glycol, and ~125° for *n*-hexadecane. There is no significant difference between the three soft PDMS types. The CAs of all liquids are significantly higher on the replicated samples compared to the samples polymerized under air. However, all liquids pin on the surfaces as well and show no ROAs. To introduce an additional microstructure on the surface, Fluoropor 35-70 replicas were prepared from wrinkled PF-PDMS (PF-SF, PF-Bioclear B, and PF-Bioclear C) molds. The wrinkled Fluoropor 35-70 replicates show higher CAs of >165° for water, >155° for ethylene glycol, and ~135° for *n*-hexadecane. Additionally, water and ethylene glycol roll-off the surfaces at ROA values below 10° and ~21°, respectively (see Figure 2A,B). In conclusion, a significant enhancement of the wetting properties was achieved by replication from wrinkled PDMS surfaces. 

**Figure 3:**
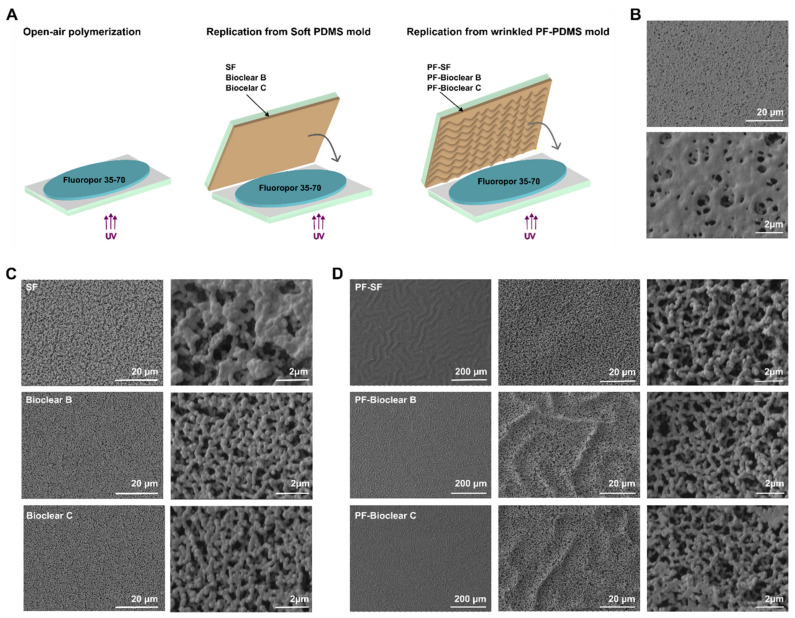
SEM images of different Fluoropor 35-70 surfaces prepared by different replication techniques. (**A**) Schematic showing the preparation process to fabricate the different Fluoropor 35-70 surfaces prepared under air, against smooth PDMS, and against wrinkled PDMS. (**B**) SEM image of the open-air-polymerized surface showing a mostly closed surface. SEM images of the replicated Fluoropor 35-70 surfaces from (**C**) smooth PDMS surfaces and (**D**) SEM images of the replicated Fluoropor 35-70 surfaces showing the replicated porous wrinkles and an open porous structure.

**Figure A3:**
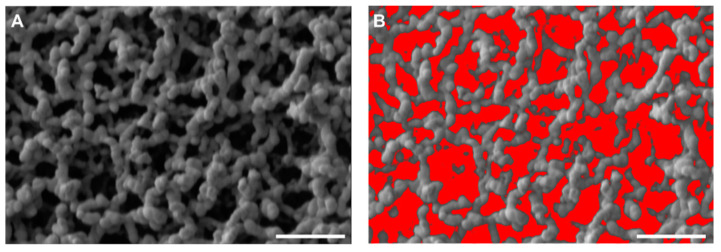
Determination of the surface porosity: Image processing analysis obtained from ImageJ for the determination of the porous area. (**A**) SEM image of the Fluoropor 35-70 replicated from PF-SF mold (8-bit image). (**B**) The pores/voids were selected by adjusting the threshold value (colored in red). Subsequently, the function “analyze particles” was applied to determine and outline the voids. The single pixels that are not related to the pores/voids were excluded by setting a lower size margin. Finally, the surface porosity values were calculated. Scale bar: 2 µm.

To investigate the observed wetting behavior of the surfaces, the surfaces structures of the samples were analyzed with SEM (see Figure 3). The surface polymerized under air shows less porosity than the surfaces replicated against smooth PDMS types of SF, Bioclear B, and Bioclear C (Figure 3B,C). This inhomogeneity in the porous network explains the CAs < 150° and poor dewetting behavior of the surfaces polymerized under air. The surfaces of Fluoropor replicated against soft PDMS are shown in Figure 3C. The porosity of SF surfaces differs slightly from the Bioclear B and C surfaces: In a close-up, it appears that in some areas of the Fluoropor replicated from SF, the pores are covered with a layer of nonporous material. However, overall, this partial nonporosity does not affect the wetting behavior, as, presumably, the roughness of the surfaces is still high enough to cause enhanced wetting properties. The Fluoropor surfaces replicated from wrinkled PF-PDMS show both the wrinkled structure and micro-/nanostructure (see Figure 3D). The wrinkled Fluoropor surfaces show an open porous network, which was successfully achieved by supercritically drying the samples. The replicated wrinkles match up well to the prepared PF-PDMS molds, where both Bioclear replicas show smaller and narrow wrinkles, whereas PF-SF replicas show larger wrinkles. To further investigate this, the surface porosity was determined by image analysis using ImageJ (see Figure A3 and Table A2). To determine surface porosity, the area fraction of the voids in the images was analyzed: Fluoropor surfaces replicated from PF-PDMS show the highest surface porosity percentage of 46–48%, followed by the surfaces replicated from smooth PDMS with 30–37% porosity, and lastly, the open-air-polymerized Fluoropor surfaces with 13% porosity. 

Overall, the introduction of a new pattern (wrinkles) enabled the achievement of superhydrophobic surfaces with a maintained surface porosity and enhanced both the wetting and dewetting properties of the replicated Fluoropor 35-70 surfaces. 

## 4. Conclusions

In summary, we present the fabrication of superhydrophobic surfaces with an enhanced liquid repellence, using a highly fluorinated polymer, via direct replication. The templates/molds for the direct replication were wrinkled surfaces, which were prepared by depositing Parylene F onto soft PDMS substrates. The replicated wrinkled porous surfaces showed an open micro-/nanoporous network without further need of any surface treatment to expose it. This was achieved using the supercritical drying approach, which helped preserve the micro-/nanoporous network at the surfaces and prevented its collapse. The replicated wrinkled Fluoropor surfaces showed a high water CA of >165° and a low ROA of <10°. Altogether, the introduction of a second structure to the inherent micro-/nanostructure of Fluoropor resulted in enhanced liquid repellence. The replication from easily prepared wrinkled surfaces, thus, offers an interesting possibility for enhancing the wetting properties of porous materials. 

## Figures and Tables

**Table 1:** Reactants used for the preparation of Bioclear PDMS.

PDMS Substrates	Weight (g)		
	XG 0677	DMS V31	HMS 151
Bioclear-B	9.0	4.5	1.5
Bioclear-C	6.4	-	2.2

**Table 2:** Measured roughness values of the PDMS and PF-PDMS surfaces with different thicknesses.

	R_q_ (nm)		
	PDMS	PF-PDMS (50 µm)	PF-PDMS (250 µm)
SF (50:1)	7	103 ± 7	110 ± 23
Bioclear-B	5	657 ± 34	487 ± 17
Bioclear-C	11	915 ± 25	815 ± 13

## Data Availability

The data is contained within the article.

## Appendix A

The surface free energy of the fabricated PF-PDMS molds was calculated according to the Owens, Wendt, Rabel, and Kaelble (OWRK) method using three liquids with different surface tensions: water, ethylene glycol, and diiodomethane [62].

**Table A1:** Surface free energy of the Parylene-F-coated PDMS templates.

	Wrinkled Templates		
	PF-SF	PF-Bioclear B	PF-Bioclear C
Surface free energy (mN/m)	25 ± 1	29 ± 1	28 ± 1

**Table A2:** Surface porosity percentage determined by image analysis using ImageJ.

	SF	PF-SF	Bioclear B	PF-Bioclear B	Bioclear C	PF-Bioclear C	Air
Porosity %	30 ± 1	47 ± 4	36 ± 2	46 ± 2	37 ± 3	48 ± 5	13 ± 1
